# Tissue Engineering of Cartilage; Can Cannabinoids Help?

**DOI:** 10.3390/ph3092970

**Published:** 2010-09-06

**Authors:** Aoife Gowran, Katey McKayed, Manoj Kanichai, Cillian White, Nissrin Hammadi, Veronica Campbell

**Affiliations:** 1 Trinity College Institute of Neuroscience and Department of Physiology, School of Medicine, University of Dublin, Trinity College, Dublin 2, Ireland; 2 Trinity Centre for Bio-Engineering, School of Engineering, University of Dublin, Trinity College, Dublin 2, Ireland

**Keywords:** phytocannabinoids, cannabinoid receptors, mesenchymal stem cells, osteoarthritis, cartilage

## Abstract

This review discusses the role of the cannabinoid system in cartilage tissue and endeavors to establish if targeting the cannabinoid system has potential in mesenchymal stem cell based tissue-engineered cartilage repair strategies. The review discusses the potential of cannabinoids to protect against the degradation of cartilage in inflamed arthritic joints and the influence of cannabinoids on the chondrocyte precursors, mesenchymal stem cells (MSCs). We provide experimental evidence to show that activation of the cannabinoid system enhances the survival, migration and chondrogenic differentiation of MSCs, which are three major tenets behind the success of a cell-based tissue-engineered cartilage repair strategy. These findings highlight the potential for cannabinoids to provide a dual function by acting as anti-inflammatory agents as well as regulators of MSC biology in order to enhance tissue engineering strategies aimed at cartilage repair.

## 1. Introduction

With significant advances in research into the role of cannabinoids in bone metabolism [1,2,3], this review endeavors to establish if there is potential for a therapeutic use of cannabinoids in tissue-engineered strategies aimed at ameliorating cartilage damage and facilitating cartilage repair. Due to the low capacity of cartilage for repair and the increasing prevalence of cartilage diseases, the tissue engineering of cartilage has become an important area of research in regenerative medicine [4,5]. The literature highlights the anti-inflammatory effects of cannabinoids and the positive influence of this on protecting against cartilage degradation in inflamed arthritic joints [6,7,8,9,10,11]. Our experimental results presented here show that activation of the cannabinoid system enhances the survival, migration and chondrogenic differentiation of the chondrogenic precursors, mesenchymal stem cells. Our data highlight the potential for the application of cannabinoids during tissue-engineered cartilage repair strategies since we show that drugs which target the cannabinoid system modulate the 3 main factors determining the success of many cartilage regeneration approaches. 

### 1.1. Tissue Engineering

Reddi defines tissue engineering as “the science of design and manufacture of new tissues for the functional restoration of impaired organs and replacement of lost parts due to disease, trauma or tumours” [12]. Furthermore, a tissue-engineered biological substitute should resemble the native tissue and perform similar biological functions [13]. Three key ingredients for the success of tissue engineering are inductive signals, responsive candidate cells, and an appropriate extracellular matrix (ECM). Inductive signals, such as growth factors, are an essential component to any tissue-engineered substitute since most candidate cells used in tissue engineering have lost their intrinsic differentiation program [13]. Hence, the identification of growth factors or other stimuli controlling the differentiation process is of crucial importance and the cannabinoid system may represent a novel target in this regard. A variety of candidate cells are used in tissue engineering, including chondrocytes, osteoblasts, embryonic stem cells and adult stem cells. The application of adult stem cells in tissue engineering holds great promise since there are no associated ethical problems with their use and they surpass the growth and differentiation capacity of native cells such as chondrocytes. The ECM is comprised of structural and specialized proteins combined with proteoglycans that form a microenvironment for cells to reside in, cells which in turn contribute to its formation. Each of the three ingredients for the success of tissue engineering should encourage cell migration, survival and proliferation, and precursor differentiation which are the three main tenets for the success of any tissue-engineered approach to regeneration [14,15]. Many challenges lie ahead in the path to the use of tissue-engineered approaches in the clinic namely the control of differentiation, immunologic issues, tissue integration and the consistency of engineered tissues. Understanding more about what controls these facets of tissue regeneration will be of the upmost importance for the generation of better tissue-engineered approaches and products. 

### 1.2. Cartilage Disease

Chondrocytes are responsible for maintaining cartilage tissue homeostasis by balancing the biosynthesis and incorporation of extracellular matrix components. Cartilage has poor to no blood supply and derives nutrients from the surrounding synovial fluid [16,17,18]. Therefore, chondrocytes are continually challenged by a hypoxic cellular micro-environment, as low as 1% oxygen during endochondral ossification [19,20,21]. Cartilage-specific extracellular matrix components, such as collagen II and sulphated proteoglycans, are synthesized by chondrocytes in order to maintain cartilage homeostasis [22,23,24]. This balance may be destroyed, however during injury or disease, resulting in a loss of cartilage and joint damage. Since cartilage is poorly supplied by blood vessels, nerves and the lymphatic system, its regenerative capacity is limited which can prove problematic. Damage as a result of the inflammation associated with joint diseases such as osteoarthritis, or traumatic injury can lead to pain and long-term disability. There are many diseases that affect cartilage the majority of which are rare, e.g., polychondritis and achondroplasia. However, changing lifestyles and an ageing global population has led to increased levels of ‘wear and tear’ diseases of cartilage such as intervertebral disc herniation and trauma-induced tears, and ‘failure to repair’ diseases of cartilage such as osteoarthritis. Bio-engineering applications are currently enhancing the treatment of cartilage disease by providing both *in situ* regeneration and cell and tissue transplantation [15]. However, identifying factors that may enhance the survival and differentiation of transplanted and resident cells warrants investigation.

A cell based tissue engineering approach to cartilage disease has the potential to become the superior treatment choice, providing biological resurfacing of affected joints [25] compared to mechanical approaches such as total joint arthroplasty or *in situ* cartilage regeneration techniques such as the microfracture technique and autologus chondrocyte transplantation (ACT) [26,27]. Some success is noted with these techniques however, long recovery periods, the slow regeneration of cartilage and lack of recipient response (especially older patients) make the search for better alternatives particularly relevant [28,29,30]. Furthermore, osteoarthritis is an exclusion criteria for ACT and the microfracture technique [31].

### 1.3. Cannabinoid System

For many centuries, cannabis has been used recreationally, as a result of its psychoactivity, mainly produced by Δ^9^-tetrahydrocannabinol (Δ^9^-THC), and medicinally for the treatment of many medical ailments, including nausea, pain, migraine, epilepsy, glaucoma and hypertension [32]. The cannabinoid system consists of cannabinoid receptors (CB) types 1 and 2, endogenous cannabinoids (endocannabinoids; namely anandamide and 2-arachidonoyl glycerol), and the enzymes that synthesize and degrade endocannabinoids. Additionally, the endocannabinoids noladin ether, *N*-arachidonyl dopamine and virodhamine, and the *N*-acyl ethanolamine palmitoylethanolamide may also have potential therapeutic effects [33]. CB_1_ receptors are found predominantly at central and peripheral nerve terminals where they mediate inhibition of transmitter release [34]. Their distribution pattern within the central nervous system accounts for several characteristic effects of CB_1_ receptor agonists, including their ability to produce hypokinesia and catalepsy and to induce signs of analgesia in both animals and man [33,35]. CB_2_ receptors occur mainly on immune cells, and may likely be involved in the modulation of cytokine release and immune cell migration [36]. Although often regarded as peripheral receptors, CB_2_ receptors have been detected in the central nervous system on microglia and neurones [37]. In addition, there are alternative targets which endocannabinoids can mediate their pharmacological actions e.g., orphan G-protein coupled receptors, peroxisome proliferator-activated receptors and transient receptor potential channels [38]. Finally, emerging evidence implicates cannabinoids in a wide variety of physiological and pathophysiological processes from skeletal maintenance [3,39] to neurodegenerative disorders [40]. 

## 2. Tissue Engineering Strategies to Solve Cartilage Disease

One particular consequence of the ageing process in humans is the inevitable impaired locomotion due to bone and joint problems arising from lack of endogenous repair mechanisms. Among the many tissues in the body, bone has some potential for repair, however cartilage lacks effective endogenous repair mechanisms, thus making it vulnerable to damage due to ‘wear and tear’ or an underlying pathology ‘failure to repair’. Chondrocytes serve to maintain cartilage tissue homeostasis, maintaining the crucial balance between the rate of biosynthesis and incorporation of matrix components, and the rate of their degradation and subsequent loss from the cartilage into the synovial fluid [41]. Active proteinases such as aggrecans, collageneses and matrix metalloproteinases (MMPs) become involved during cartilage resorption, degrading major components of the cartilage extracellular matrix, such as collagen type II and proteoglycans (mainly aggrecan) [42]. These proteinases are secreted from the cells in a latent form, requiring extracellular activation, and are inhibited by tissue inhibitors of metalloproteinases (TIMPs) [43]. An imbalance between these TIMPs and proteinases accounts for some of the cartilage destruction found in rheumatic conditions [42,43]. 

Many techniques are currently employed to treat cartilage damage, however none are satisfactory in the long term. Although appearing to be a simple tissue composed of only a single cell type and extracellular matrix researchers have yet to design and produce a tissue akin to articular cartilage that can resist compression and distribute loads as effectively [44,45,46]. Due to the complexity of cartilage physiology the most suitable method for the treatment of damaged cartilage would be to promote natural regeneration or biological resurfacing [25]. The use of mesenchymal stem cells (MSCs) to tissue engineer new cartilage offers potential, and in combination with cannabinoid drugs could provide an alternative solution to the treatment of cartilage disease. 

### 2.1. Bone Marrow Derived Mesenchymal Stem Cells and Chondrogenesis

Post-natal bone marrow is an organ composed of two main systems rooted in distinct lineages; the haematopoietic tissue proper and the associated supporting stromal cells or mesenchymal stem cells (MSCs). Originally examined because of their critical role in the formation of the haematopoietic microenvironment, mesenchymal stem cells (MSCs) later came to centre stage with the recognition that they are the stem/progenitor cells of skeletal tissues [47,48,49]. While they represent a minor fraction of the total nucleated cell population in marrow (0.001%–0.01%), MSCs can be plated and enriched using standard cell culture techniques [50,51]. MSCs have also been isolated from other tissues such as adipose [52], synovial membrane [53] and skeletal muscle [54]. The differentiation of MSCs into bone, cartilage and fat has been described and characterized by multiple laboratories [50,55,56,57,58,59]. Data pointing to the unexpected differentiation potential of MSCs into neural tissue may potentially grant them membership into the diverse family of putative adult pluripotent stem cells [60]. MSCs migrate to sites of tissue damage and modulate the inflammatory and survival status of cells within the site of damage due their ability to downregulate pro-inflammatory cytokines and produce anti-inflammatory and pro-survival factors [61]. Despite this knowledge several aspects of MSC cell biology remain to be elucidated including (1) their exact identity (no one specific MSC marker or set of specific MSC markers have been determined to date); (2) their developmental origin and *in vivo* niche and (3) their amenability to *ex vivo* manipulation and *in vivo* use in the clinic [62]. 

MSC chondrogenic differentiation generally occurs when culture-expanded MSCs are grown under conditions that include (1) a 3-D culture format; (2) a serum-free nutrient medium and (3) the addition of a member of the TGF-β super-family. We have demonstrated that treatment of MSCs with ascorbic acid, dexamethasone and TGF-β1 induces chondrogenesis [63,64]. When these conditions are met the cells rapidly lose their fibroblastic morphology and begin to initiate expression of a number of cartilage-specific extracellular matrix components [55,65]. During the early stage of MSC chondrogenesis there is also a progressive change in the expression of collagen II protein and deposition of proteoglycans in the extracellular matrix. Furthermore, the rapid biosynthesis of glycosaminoglycan (GAG; a polysaccaride forming an important part of connective tissues) is accompanied by dramatic alterations in chondrocyte morphology [66]. The isolation of chondrocytes from their reparative MSC precursors, coupled with the challenges associated with current cartilage repair strategies involving cell/tissue transplantation, makes the *in situ* recruitment of MSCs to the area of damage a more attractive avenue of research [15]. Additionally MSCs have notable anti-inflammatory properties which may be beneficial in inflamed joints [67]. The potential for the cannabinoid system to control inflammation, MSC migration and chondrogenesis could be beneficial to diseased cartilage tissue and will now be discussed in the following sections.

## 3. Cannabinoid System Potential Use in Tissue-Engineered Cartilage

### 3.1. Cannabinoid System and Chondrogenesis

Our review of the available literature on the role of cannabinoids in chondrogenesis revealed a caveat in the research particularly concerning the chondrocyte precursors and the process of chondrogenesis. The role of the cannabinoid system in primary chondrocytes and cartilage explant cultures has been investigated [6,7]. Mdvundula and co-workers have studied the effects of cannabinoids on the production of nitric oxide, which is produced in large quantities by chondrocytes from L-arginine oxidation by inducible nitric oxide synthase (iNOS) when stimulated by interleukin-1 (IL-1) or lipopolysaccharide (LPS). Their study investigated whether or not cannabinoids have an effect on chondrocyte metabolism, thus leading to a reduction in cartilage breakdown, thereby forming a basis for the identification of novel potential anti-arthritic drugs. Their results showed that the synthetic cannabinoids, Win-55,212-2 and HU-210, inhibited IL-1α-induced nitric oxide (NO) production and both anandamide and Win-55,212-2 inhibited IL-1α-induced proteoglycan degradation in bovine articular chondrocytes [6,7]. Studies utilizing cannabinoid receptor antagonists, AM281 (CB_1_) and AM630 (CB_2_), showed that when employed on their own, they reduced NO production. Furthermore, instead of inhibiting the effect of Win-55,212-2 on NO production they appeared to act synergistically when applied in combination with the cannabinoid [6]. This effect is probably due to the inverse agonist activity observed when antagonists are used at high concentrations like those used in the Mdvundula and co-workers studies (5-100 μM) [6,7,68] or the antagonists were acting via receptors other than cannabinoid receptors such as GPR55 or PPARγ, or were receptor-independent. Such uncertainty made it impossible to tell if the effect of Win-55,212-2 was mediated through either the CB_1_ or CB_2_ receptor. Mbvundula and co-workers concluded that chondrocytes constitutively express cannabinoid receptors and propose that synthetic cannabinoids protect cartilage matrix degradation induced by increased levels of cytokines and NO metabolism during inflammation [6,7]. 

The cannabinoid system is present in ancillary cells and tissues to cartilage such as synoviocytes, myofibroblasts, fibroblasts and MSCs [10,69,70]. Indeed, the non-psychoactive cannabinoid, ajulemic acid suppresses the degradation of cartilage induced by fibroblastic metalloproteinases [9]. Degradation of cartilage is a pathological feature of rheumatoid arthritis and there is evidence to suggest that targeting the cannabinoid system may be a potential treatment for ameliorating the symptoms of conditions that involve cartilage degradation [11,71,72,73,74,75]. For example ajulemic acid selectively increases anti-inflammatory eicosanoid production and greatly reduces permanent joint damage [72,73,75]. Additionally, another non-psychoactive phytocannabinoid, cannabidiol displays combined immunosuppressive and anti-inflammatory actions in murine collagen-induced arthritis [74]. Furthermore, upregulation of the cannabinoid system has been observed in synovial tissue and fluid taken from patients with osteoarthritis and rheumatoid arthritis [10], an additional indicator that the cannabinoid system has a role in the maintenance of joint homeostasis in rheumatic conditions. 

Although only limited research on the role of the cannabinoid system in MSC biology has been conducted [39,70] there is adequate ancillary research pointing to the conclusion that such an avenue of research should be pursued. In John McPartland’s thought provoking 2008 review on the expression of the endocannabinoid system in myofascial tissues and fibroblasts [69] he raises the idea that the endocannabinoid system may be as influential in skeletal tissue repair as it is in neurogenesis [76] and brain development [77] since “the forces of embryogenesis become the forces of healing after birth” [78]. Therefore, we propose that there is a potential role for the cannabinoid system in tissue-engineered regeneration strategies. 

## 4. Is the Cannabinoid System a Potential Therapeutic Target in MSC-Based Tissue-Engineered Cartilage Regeneration Strategies?

**Figure 1:**
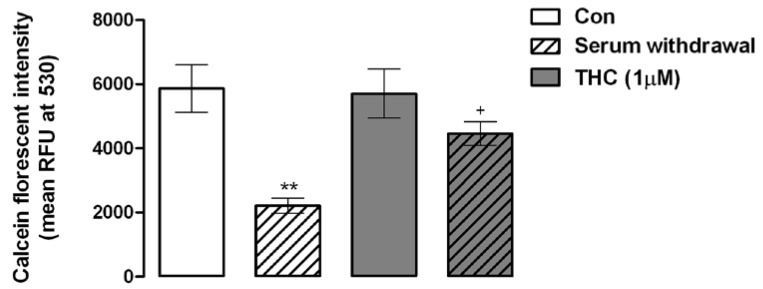
The phytocannabinoid, Δ^9^-THC enhances the survival of MSCs during cellular stress. Serum withdrawal significantly reduced the metabolic function of MSCs compared to control MSCs (Con; **p < 0.01, 1-way ANOVA and Newman-Keuls, *n* = 4) as assessed by the ability of MSCs to metabolize the fluorescent substrate calcein AM. Treatment of MSCs with Δ^9^-THC (1 μM, 24 hours) protected MSCs from serum withdrawal compared to serum deprived MSCs (Serum withdrawal; +*p* < 0.05, 1-way ANOVA and Newman-Keuls, *n* = 4).

Since there was a caveat in the research concerning the role of the cannabinoid system in MSC chondrogenesis we conducted experiments to assess if the cannabinoid system influenced cartilage precursor cells (MSCs). We examined three facets of MSC cell biology: survival, migration and differentiation which are stipulated as being crucial tenets for MSC-based *in situ* tissue regeneration strategies [15]. We found that activation of the cannabinoid system protected MSCs during the acute stress of serum withdrawal; an *in vitro* simulation of the hostile environment of the inflamed joint (Figure 1). In control MSCs calcein fluorescent intensity, a marker of cellular metabolism and viability, was 5.9 ± 0.74 (× 10^4^ RFU, mean ± SEM) and this was significantly reduced to 2.2 ± 0.23 following serum withdrawal for 24 hours (**p < 0.01, 1-way ANOVA and Newman-Keuls, *n* = 4; Figure 1). When MSCs were exposed to serum withdrawal in the presence of Δ^9^-THC (1 μM, 24 hours), calcein fluorescence was unaffected, indicating that Δ^9^-THC protected MSCs against serum withdrawal (*p* < 0.05, 1-way ANOVA and Newman-Keuls, *n* = 4; Figure 1). MSCs expanded in culture for 3 weeks and exposed to Δ^9^-THC alone (1 μM; 24 hours) displayed no changes in viability. However, previous results in our laboratory have demonstrated that exposure to Δ^9^-THC (1 μM, 24 hours) decreased MSC survival in MSCs cultured for 5 weeks (unpublished observations). This indicates that there is a temporal aspect to the outcome of the activation of the cannabinoid system in MSCs possibly resulting from age-differences in the propensity towards senescence. Thus, the temporal response of cannabinoid system activation in MSCs that we have observed could represent an important aspect of MSC cell biology that could be exploited to enhance cell-based regenerative strategies. 

**Figure 2:**
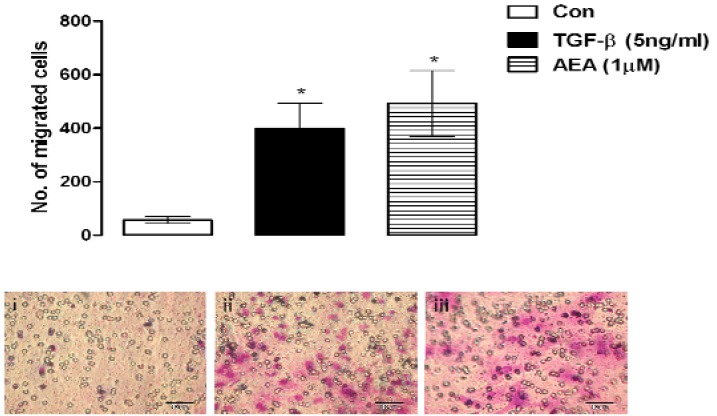
MSCs migrate towards the endocannabinoid anandamide. TGF-β (5 ng/mL; 48 hours) induced a significant increase in the migration of MSCs as assessed by the Boyden chamber cell migration assay compared to the number of MSCs that migrated to control media (Con; *p < 0.05, 1-way ANOVA and Newman-Keuls, *n* = 3). Migration of MSCs was also induced by anandamide (AEA; 1 μM, 48 hours) compared to control (Con; *p < 0.05, 1-way ANOVA and Newman-Keuls, *n* = 3). Inset: images of MSCs that migrated towards (i) control media; (ii) TGF-β (5 ng/mL; 48 hours) and (iii) AEA (1 μM, 48 hours).

Enhancing the number of MSCs migrating to the site of tissue injury is a means to expedite regeneration and improve end results [15] therefore any factors that influence this facet of MSC biology may have the potential to enhance the success of cell-based tissue-engineered regenerative strategies. Therefore we assessed the chemo-attractant effect of the endocannabinoid, anandamide (AEA; 1 μM, 48 hours) on MSCs using the Boyden chamber cell migration assay (Figure 2). Anandamide had a pro-migratory effect on MSCs inducing a significant 7 fold increase in the number of migrating cells compared to control media (Con; *p* < 0.05, 1-way ANOVA and Newman-Keuls, *n* = 3 Figure 2). This was comparable to the effect induced by TGF-β a known inducer of MSC migration [79]. Thus, the endocannabinoid, anandamide has a positive influence on MSC migration providing enhanced repair potential which may accelerate tissue regeneration.

**Figure 3:**
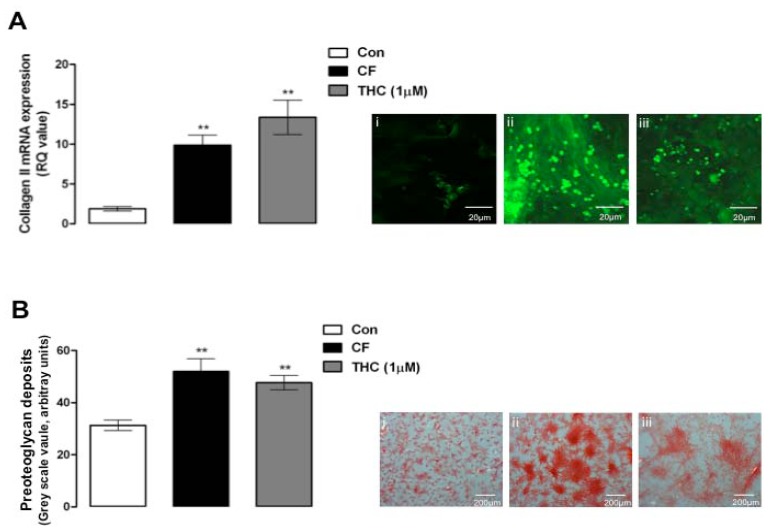
Δ^9^-THC enhances MSC chondrogenesis. A, chondrogenic factors (CF) induced a significant increase in collagen II mRNA expression as assessed by qPCR after 2 weeks of treatment compared to control MSCs (Con; **p = 0.01, 1-way ANOVA and Newman-Keuls, *n* = 3). Δ^9^-THC (1 μM, 2 weeks) also enhanced chondrogenesis compared to control MSCs (**p = 0.01, 1-way ANOVA and Newman-Keuls, *n* = 3). Inset: images of collagen protein expression in MSCs exposed to control conditions (i) CF for 2 weeks; (ii) and Δ^9^-THC (1 μM) for 2 weeks; (iii) B, chondrogenic factors (CF) induced a significant increase in proteoglycan deposits in the extracellular matrix as assessed by safranin-O staining after 2 weeks of treatment compared to control MSCs (Con; **p = 0.01, 1-way ANOVA and Newman-Keuls, *n* = 4). Δ^9^-THC (1 μM, 2 weeks) also enhanced proteoglycan deposition compared to control MSCs (**p = 0.01, 1-way ANOVA and Newman-Keuls, *n* = 4). Inset: safranin-O stained MSCs exposed to control conditions (i) CF for 2 weeks; (ii) and Δ^9^-THC (1 μM) for 2 weeks.

Finally we assessed the effect of Δ^9^-THC on MSC chondrogenesis by measuring collagen II expression and the deposition of proteoglycans in the extracellular matrix. MSCs were shown to differentiate into chondrocytes as determined by qPCR analysis of collagen II mRNA and protein expression, and the presence of proteoglycan deposits in the extracellular matrix (**p = 0.01, 1-way ANOVA and Newman-Keuls, *n* = 3, Figure 3). Treatment of MSCs with Δ^9^-THC enhanced the chondrogenesis of MSCs as shown by increased levels of collagen II mRNA and protein expression, and increased levels of proteoglycan deposits (**p = 0.01, 1-way ANOVA and Newman-Keuls, *n* = 4; Figure 3).

## 5. Conclusions and Future Directions

Although knowledge of the role of the cannabinoid system on bone and osteogenesis is well documented, and continues to become further insightful, the role of the cannabinoid system in cartilage tissue has lagged behind. This review aimed to assess the potential application of cannabinoids in enhancing MSC-based tissue-engineered cartilage regeneration strategies. Overall we have shown that modulation of the cannabinoid system can affect the major facets of MSC cell biology: survival, migration and differentiation. We conclude that there is sufficient evidence from the past literature and from our experimental evidence to support the potential of cannabinoid-based drugs in tissue-engineered applications aimed at reducing cartilage degradation and facilitating cartilage repair. 

## 6. Methods

### 6.1. Culture of Mesenchymal Stem Cells

Three-month old Wistar rats (250 g-300 g) were obtained from the Bioresources Unit, University of Dublin, Trinity College. Animals were sacrificed by CO_2_ asphyxiation or cervical dislocation in accordance with European guidelines (86/609/EEC). The femur was dislocated from the tibia and placed in sterile pre-warmed Dulbecco’s modified Eagle’s medium (DMEM; Sigma-Aldrich, England) supplemented with 10% foetal bovine serum; 100 U/mL penicillin/streptomycin; 2 mM glutamax; 1 mM L-glutamine and 1% non-essential amino acids (s-DMEM; Invitrogen, Scotland). The femur and tibia were cut at both epiphyses and bone marrow was flushed into a 50 mL tube using 5 mL s-DMEM and a 25-gauge needle. The suspension was centrifuged (650 g) for 5 minutes at 20^o^C, resuspended in 10 mL of s-DMEM and passed sequentially through 16-, 18- and 20-guage needles. The suspension was passed through a 40 μm nylon mesh into a sterile Petri dish and incubated in a humidified atmosphere (95% air and 5% CO_2_) at 37^o^C for 30 min. The supernatant was removed and split between two T75 flasks. Culture media was replaced following 24 hours to remove non-adherent cells. Cells were passaged upon reaching 80%-90% confluency to a maximum of 4 passages. The medium was replaced every 3 to 4 days. To induce chondrogenesis cells were treated with chondrogenic factors (CF); 100 nM dexamethasone, 50 μM ascorbic acid and 5 ng/mL TGF-β for the indicated time period. 

### 6.2. Drug Treatments

MSCs were incubated with drugs or vehicle for the time indicated in each experiment. Δ^9^-THC and anandamide were obtained from Sigma-Aldrich Company Ltd. Δ^9^-THC was held under license granted by the Irish Department of Health and Children. Δ^9^-THC was stored as a 80mM stock solution in ethanol at -20^o^C and diluted to a final concentration of 1 μM in culture media. Anandamide was stored as a 10 mM stock solution in ethanol at -20^o^C and diluted to a final concentration of 1 μM in culture media.

### 6.3. Cell Viability Assay

Cell viability was determined by quantifying the enzymatic conversion of cell permeable calcein AM (Invitrogen, Scotland) to a fluorescent product by active intracellular esterases. Briefly, MSCs were grown on sterile 96 well plates (6 × 10^3^ cells per well) and treated as indicated in each experiment. MSCs were washed in pre-warmed PBS to remove serum from the wells. Calcein AM solution (2 μM in PBS) was applied to each well and incubated in a humidified atmosphere (95% air and 5% CO_2_) at 37 ^o^C for 1 hour. Following incubation calcein fluorescence at 530 nm was determined using a microplate reader heated to 37^o^C (Synergy™ HT, BioTek Instruments, USA). Data are presented as mean relative fluorescent intensity units (RFU) ± SEM. 

### 6.4. Cell Migration

For cell migration experiments 50 × 10^3^ cells in s-DMEM were seeded onto the upper chamber of a migration chamber (8 μm Millicell 24 well plate cell culture insert, Millipore, Ireland) and s-DMEM was added to the bottom chamber. Cells were allowed to settle for 48 hours before beginning migration experiments. Chemoattractant media (5 ng/mL TGF-β; + control), anandamide (1 μM) or control non chemoattractant media (DMEM supplemented with 1% BSA, 100U/mL penicillin/streptomycin; 2 mM glutamax; 1 mM L-glutamine and 1% non-essential amino acids) were placed into the bottom chamber and cells were allowed to migrate for 48 hours. Cells remaining on the upper chamber were scrapped off whilst cells that had migrated to the lower chamber were stained using the Rapi Diff II staining kit (Bios Europe, UK). Blinded scorers manually counted cells from 10 random fields at 20 × magnification. Data are presented as mean number of migrated cells ± SEM.

### 6.5. Real-time PCR for Collagen II

Total RNA was isolated from MSCs using a NucleoSpin^®^ total RNA isolation kit (Macherey-Nagel Inc., Germany) following the manufacturer’s instructions. This protocol included a DNase step in order to remove any genomic DNA contamination. Total RNA concentrations were determined by spectrophotometry (NanoDrop Technologies, USA) and stored at -80^o^C until required for cDNA synthesis. Total RNA concentrations were adjusted to a standard concentration prior to cDNA synthesis. cDNA was generated from 0.5-1 μg total RNA using High Capacity cDNA Archive kit (Applied Biosystems, Germany) following the manufacturer’s instructions. The resultant cDNA was stored at -20^o^C until required for real time PCR. Real-time PCR was performed using Taqman Gene expression assays (Applied Biosystems, Germany) on an ABI Prism 7300 instrument (Applied Biosystems, Germany). The assay ID for the collagen II gene was Rn01637085 mL. Rat β-actin (4352340E) was used as an endogenous control. Gene expression was calculated relative to the endogenous control (β-actin) and to the control samples to give a relative quantification (RQ) value. 

### 6.6. Immunofluorescence for Collagen II

Following drug treatment MSCs were fixed in 100% methanol for 5 minutes at -20^o^C, permeabilized with 0.2% Triton-X100 for 10 min and washed in three changes of PBS at room temperature (RT). Cells were washed 3×5 min in PBS and non-reactive sites were blocked with blocking buffer (2% BSA in PBS with 0.1% Triton × 100 and 20% heat-inactivated horse serum; Vector, USA) for two hours at room temperature (22^o^C). The cells were incubated overnight with mouse anti-collagen II antibody (1:500 dilution in blocking buffer; Abcam, Cambridge, UK) at 4^o^C. Cells were washed 3 × 5 min in PBS, incubated with a biotinylated secondary horse anti-mouse IgG (1:50 dilution; Vector, USA) for one hour at room temperature and washed 3 × 5 min in PBS. In a light protected environment, Extravidin FITC (1:50 dilution in PBS; Sigma-Aldrich, England) was added for one hour washed 6 × 5 min in dH_2_O. Coverslips were mounted onto glass slides in Vectashield mounting medium (Vector, USA) and sealed using nail varnish. Slides were examined at 20 × magnification with a fluorescence microscope (Zeiss, Germany). 

### 6.7. Safranin-O Histological Staining for Proteoglycans

Fixed cells on coverslips were washed with PBS for 5 min, stained with Safranin-O stain (1% in dH_2_O; Sigma-Aldrich, England) for 25 min then washed four times in dH_2_O. Cells were examined at 20 × magnification using an inverted microscope (Olympus) and digital images were acquired using Analysis-D software. Images were imported into UTHSCA ImageTool (version 3.00, The University of Texas Health Science Centre, USA). Threshold values were set and converted to a binary image before gray scale value quantification. Data are presented as mean grey scale value ± SEM. 

## References

[1] Idris A.I., van 't Hof R.J., Greig I.R., Ridge S.A., Baker D., Ross R.A., Ralston S.H. (2005). Regulation of bone mass, bone loss and osteoclast activity by cannabinoid receptors. Nat. Med..

[2] Ofek O., Karsak M., Leclerc N., Fogel M., Frenkel B., Wright K., Tam J., Attar-Namdar M., Kram V., Shohami E., Mechoulam R., Zimmer A., Bab I. (2006). Peripheral cannabinoid receptor, CB2, regulates bone mass. Proc. Natl. Acad. Sci. USA.

[3] Idris A.I., Ralston S.H. (2010). Cannabinoids and Bone: Friend or Foe?. Calcif. Tissue Int..

[4] Ahrens P.B., Solursh M., Reiter R.S. (1977). Stage-related capacity for limb chondrogenesis in cell culture. Dev. Biol..

[5] Guilak F., Awad H.A., Fermor B., Leddy H.A., Gimble J.M. (2004). Adipose-derived adult stem cells for cartilage tissue engineering. Biorheology.

[6] Mbvundula E.C., Bunning R.A., Rainsford K.D. (2005). Effects of cannabinoids on nitric oxide production by chondrocytes and proteoglycan degradation in cartilage. Biochem. Pharmacol..

[7] Mbvundula E.C., Bunning R.A., Rainsford K.D. (2006). Arthritis and cannabinoids: HU-210 and Win-55,212-2 prevent IL-1alpha-induced matrix degradation in bovine articular chondrocytes *in vitro*. J. Pharm. Pharmacol..

[8] Bidinger B., Torres R., Rossetti R.G., Brown L., Beltre R., Burstein S., Lian J.B., Stein G.S., Zurier R.B. (2003). Ajulemic acid, a nonpsychoactive cannabinoid acid, induces apoptosis in human T lymphocytes. Clin. Immunol..

[9] Johnson D.R., Stebulis J.A., Rossetti R.G., Burstein S.H., Zurier R.B. (2007). Suppression of fibroblast metalloproteinases by ajulemic acid, a nonpsychoactive cannabinoid acid. J. Cell. Biochem..

[10] Richardson D., Pearson R.G., Kurian N., Latif M.L., Garle M.J., Barrett D.A., Kendall D.A., Scammell B.E., Reeve A.J., Chapman V. (2008). Characterisation of the cannabinoid receptor system in synovial tissue and fluid in patients with osteoarthritis and rheumatoid arthritis. Arthritis Res. Ther..

[11] McDougall J.J., Yu V., Thomson J. (2008). *In vivo* effects of CB2 receptor-selective cannabinoids on the vasculature of normal and arthritic rat knee joints. Br. J. Pharmacol..

[12] Reddi A.H. (2000). Morphogenesis and tissue engineering of bone and cartilage: inductive signals, stem cells, and biomimetic biomaterials. Tissue Eng..

[13] Nesic D., Whiteside R., Brittberg M., Wendt D., Martin I., Mainil-Varlet P. (2006). Cartilage tissue engineering for degenerative joint disease. Adv. Drug Deliv. Rev..

[14] Lu L., Zhu X., Valenzuela R.G., Currier B.L., Yaszemski M.J. (2001). Biodegradable polymer scaffolds for cartilage tissue engineering. Clin. Orthop. Relat. Res..

[15] Richter W. (2009). Mesenchymal stem cells and cartilage *in situ* regeneration. J. Intern. Med..

[16] Grimshaw M.J., Mason R.M. (2000). Bovine articular chondrocyte function *in vitro* depends upon oxygen tension. Osteoarthritis Cartilage.

[17] Malda J., Martens D.E., Tramper J., van Blitterswijk C.A., Riesle J. (2003). Cartilage tissue engineering: controversy in the effect of oxygen. Crit. Rev. Biotechnol..

[18] Marcus R.E., Srivastava V.M. (1973). Effect of low oxygen tensions on glucose-metabolizing enzymes in cultured articular chondrocytes. Proc. Soc. Exp. Biol. Med..

[19] Brighton C.T., Heppenstall R.B. (1971). Oxygen tension of the epiphyseal plate distal to an arteriovenous fistula. Clin. Orthop. Relat. Res..

[20] Brittberg M., Tallheden T., Sjogren-Jansson B., Lindahl A., Peterson L. (2001). Autologous chondrocytes used for articular cartilage repair: an update. Clin. Orthop. Relat. Res..

[21] Haselgrove J.C., Shapiro I.M., Silverton S.F. (1993). Computer modeling of the oxygen supply and demand of cells of the avian growth cartilage. Am. J. Physiol..

[22] Shum L., Nuckolls G. (2002). The life cycle of chondrocytes in the developing skeleton. Arthritis Res..

[23] DeLise A.M., Fischer L., Tuan R.S. (2000). Cellular interactions and signaling in cartilage development. Osteoarthritis Cartilage.

[24] Sandell L.J., Adler P. (1999). Developmental patterns of cartilage. Front. Biosci..

[25] Goldberg V.M., Caplan A.I. (1994). Biological resurfacing: an alternative to total joint arthroplasty. Orthopedics.

[26] Mithoefer K., McAdams T., Williams R.J., Kreuz P.C., Mandelbaum B.R. (2009). Clinical efficacy of the microfracture technique for articular cartilage repair in the knee: an evidence-based systematic analysis. Am. J. Sports Med..

[27] Steadman J.R., Rodkey W.G., Briggs K.K., Rodrigo J.J. (1999). The microfracture technic in the management of complete cartilage defects in the knee joint. Orthopade.

[28] Steadman J.R., Rodkey W.G., Briggs K.K. (2002). Microfracture to treat full-thickness chondral defects: surgical technique, rehabilitation, and outcomes. J. Knee. Surg..

[29] Kon E., Delcogliano M., Filardo G., Montaperto C., Marcacci M. (2008). Second generation issues in cartilage repair. Sports Med. Arthrosc..

[30] McNickle A.G., Provencher M.T., Cole B.J. (2008). Overview of existing cartilage repair technology. Sports Med. Arthrosc..

[31] Richter W. (2007). Cell-based cartilage repair: illusion or solution for osteoarthritis. Curr. Opin. Rheumatol..

[32] Pertwee R.G. (2005). The therapeutic potential of drugs that target cannabinoid receptors or modulate the tissue levels or actions of endocannabinoids. AAPS J..

[33] Pertwee R.G. (2005). Pharmacological actions of cannabinoids. Handb. Exp. Pharmacol..

[34] Howlett A.C., Barth F., Bonner T.I., Cabral G., Casellas P., Devane W.A., Felder C.C., Herkenham M., Mackie K., Martin B.R., Mechoulam R., Pertwee R.G. (2002). International Union of Pharmacology. XXVII. Classification of cannabinoid receptors. Pharmacol. Rev..

[35] Walker J.M., Hohmann A.G. (2005). Cannabinoid mechanisms of pain suppression. Handb. Exp. Pharmacol..

[36] Goutopoulos A., Makriyannis A. (2002). From cannabis to cannabinergics: new therapeutic opportunities. Pharmacol. Ther..

[37] Onaivi E.S., Ishiguro H., Gong J.P., Patel S., Perchuk A., Meozzi P.A., Myers L., Mora Z., Tagliaferro P., Gardner E., Brusco A., Akinshola B.E., Liu Q.R., Hope B., Iwasaki S., Arinami T., Teasenfitz L., Uhl G.R. (2006). Discovery of the presence and functional expression of cannabinoid CB2 receptors in brain. Ann. NY Acad. Sci..

[38] De Petrocellis L., Di Marzo V. (2010). Non-CB1, non-CB2 receptors for endocannabinoids, plant cannabinoids, and synthetic cannabimimetics: focus on G-protein-coupled receptors and transient receptor potential channels. J. Neuroimmune Pharmacol..

[39] Scutt A., Williamson E.M. (2007). Cannabinoids stimulate fibroblastic colony formation by bone marrow cells indirectly via CB2 receptors. Calcif. Tissue Int..

[40] Fowler C.J., Rojo M.L., Rodriguez-Gaztelumendi A. (2010). Modulation of the endocannabinoid system: neuroprotection or neurotoxicity?. Exp. Neurol..

[41] Steinmeyer J., Daufeldt S. (1997). Pharmacological influence of antirheumatic drugs on proteoglycans from interleukin-1 treated articular cartilage. Biochem. Pharmacol..

[42] Cawston T. (1998). Matrix metalloproteinases and TIMPs: properties and implications for the rheumatic diseases. Mol. Med. Today.

[43] Steinmeyer J., Daufeldt S., Kalbhen D.A. (1997). The proteoglycan metabolism, morphology and viability of articular cartilage treated with a synthetic matrix metalloproteinase inhibitor. Res. Exp. Med. (Berl.).

[44] Temenoff J.S., Mikos A.G. (2000). Review: tissue engineering for regeneration of articular cartilage. Biomaterials.

[45] Buckwalter J.A., Mankin H.J. (1998). Articular cartilage: tissue design and chondrocyte-matrix interactions. Instr. Course Lect..

[46] Cohen N.P., Foster R.J., Mow V.C. (1998). Composition and dynamics of articular cartilage: structure, function, and maintaining healthy state. J. Orthop. Sports Phys. Ther..

[47] Friedenstein A.J., Piatetzky S., Petrakova K.V. (1966). Osteogenesis in transplants of bone marrow cells. J. Embryol. Exp. Morphol..

[48] Friedenstein A.J., Chailakhjan R.K., Lalykina K.S. (1970). The development of fibroblast colonies in monolayer cultures of guinea-pig bone marrow and spleen cells. Cell Tissue Kinet..

[49] Owen M. (1988). Marrow stromal stem cells. J. Cell Sci..

[50] Pittenger M.F., Mackay A.M., Beck S.C., Jaiswal R.K., Douglas R., Mosca J.D., Moorman M.A., Simonetti D.W., Craig S., Marshak D.R. (1999). Multilineage potential of adult human mesenchymal stem cells. Science.

[51] Barry F.P., Murphy J.M. (2004). Mesenchymal stem cells: clinical applications and biological characterization. Int. J. Biochem. Cell Biol..

[52] Lin G., Garcia M., Ning H., Banie L., Guo Y.L., Lue T.F., Lin C.S. (2008). Defining stem and progenitor cells within adipose tissue. Stem Cells Dev..

[53] De Bari C., Dell'Accio F., Tylzanowski P., Luyten F.P. (2001). Multipotent mesenchymal stem cells from adult human synovial membrane. Arthritis Rheum..

[54] Barry F.P. (2003). Biology and clinical applications of mesenchymal stem cells. Birth Defects Res. C Embryo Today.

[55] Barry F., Boynton R.E., Liu B., Murphy J.M. (2001). Chondrogenic differentiation of mesenchymal stem cells from bone marrow: differentiation-dependent gene expression of matrix components. Exp. Cell Res..

[56] Barry F., Boynton R., Murphy M., Haynesworth S., Zaia J. (2001). The SH-3 and SH-4 antibodies recognize distinct epitopes on CD73 from human mesenchymal stem cells. Biochem. Biophys. Res. Commun..

[57] Bruder S.P., Jaiswal N., Ricalton N.S., Mosca J.D., Kraus K.H., Kadiyala S. (1998). Mesenchymal stem cells in osteobiology and applied bone regeneration. Clin. Orthop. Relat. Res..

[58] Bruder S.P., Kraus K.H., Goldberg V.M., Kadiyala S. (1998). The effect of implants loaded with autologous mesenchymal stem cells on the healing of canine segmental bone defects. J. Bone Joint Surg. Am..

[59] Digirolamo C.M., Stokes D., Colter D., Phinney D.G., Class R., Prockop D.J. (1999). Propagation and senescence of human marrow stromal cells in culture: a simple colony-forming assay identifies samples with the greatest potential to propagate and differentiate. Br. J. Haematol..

[60] Bunting K.D., Hawley R.G. (2003). Integrative molecular and developmental biology of adult stem cells. Biol. Cell.

[61] Salem H.K., Thiemermann C. (2010). Mesenchymal stromal cells: current understanding and clinical status. Stem Cells.

[62] Bianco P., Riminucci M., Gronthos S., Robey P.G. (2001). Bone marrow stromal stem cells: nature, biology, and potential applications. Stem Cells.

[63] Kanichai M., Ferguson D., Prendergast P.J., Campbell V.A. (2008). Hypoxia promotes chondrogenesis in rat mesenchymal stem cells: a role for AKT and hypoxia-inducible factor (HIF)-1alpha. J. Cell Physiol..

[64] McMahon L.A., Reid A.J., Campbell V.A., Prendergast P.J. (2008). Regulatory effects of mechanical strain on the chondrogenic differentiation of MSCs in a collagen-GAG scaffold: experimental and computational analysis. Ann. Biomed. Eng..

[65] Sekiya I., Vuoristo J.T., Larson B.L., Prockop D.J. (2002). *In vitro* cartilage formation by human adult stem cells from bone marrow stroma defines the sequence of cellular and molecular events during chondrogenesis. Proc. Natl. Acad. Sci. USA.

[66] Chen C.W., Tsai Y.H., Deng W.P., Shih S.N., Fang C.L., Burch J.G., Chen W.H., Lai W.F. (2005). Type I and II collagen regulation of chondrogenic differentiation by mesenchymal progenitor cells. J. Orthop. Res..

[67] Pistoia V., Raffaghello L. (2010). Potential of mesenchymal stem cells for the therapy of autoimmune diseases. Expert Rev. Clin. Immunol..

[68] Ross R.A., Brockie H.C., Stevenson L.A., Murphy V.L., Templeton F., Makriyannis A., Pertwee R.G. (1999). Agonist-inverse agonist characterization at CB1 and CB2 cannabinoid receptors of L759633, L759656, and AM630. Br. J. Pharmacol..

[69] McPartland J.M. (2008). Expression of the endocannabinoid system in fibroblasts and myofascial tissues. J. Bodyw. Mov. Ther..

[70] Idris A.I., Sophocleous A., Landao-Bassonga E., Canals M., Milligan G., Baker D., van't Hof R.J., Ralston S.H. (2009). Cannabinoid receptor type 1 protects against age-related osteoporosis by regulating osteoblast and adipocyte differentiation in marrow stromal cells. Cell Metab..

[71] Whyte L.S., Ryberg E., Sims N.A., Ridge S.A., Mackie K., Greasley P.J., Ross R.A., Rogers M.J. (2009). The putative cannabinoid receptor GPR55 affects osteoclast function *in vitro* and bone mass *in vivo*. Proc. Natl. Acad. Sci. USA.

[72] Stebulis J.A., Johnson D.R., Rossetti R.G., Burstein S.H., Zurier R.B. (2008). Ajulemic acid, a synthetic cannabinoid acid, induces an antiinflammatory profile of eicosanoids in human synovial cells. Life Sci..

[73] Burstein S.H. (2000). Ajulemic acid (CT3): a potent analog of the acid metabolites of THC. Curr. Pharm. Des..

[74] Malfait A.M., Gallily R., Sumariwalla P.F., Malik A.S., Andreakos E., Mechoulam R., Feldmann M. (2000). The nonpsychoactive cannabis constituent cannabidiol is an oral anti-arthritic therapeutic in murine collagen-induced arthritis. Proc. Natl. Acad. Sci. USA.

[75] Zurier R.B., Sun Y.P., George K.L., Stebulis J.A., Rossetti R.G., Skulas A., Judge E., Serhan C.N. (2009). Ajulemic acid, a synthetic cannabinoid, increases formation of the endogenous proresolving and anti-inflammatory eicosanoid, lipoxin A4. FASEB J..

[76] Aguado T., Romero E., Monory K., Palazuelos J., Sendtner M., Marsicano G., Lutz B., Guzman M., Galve-Roperh I. (2007). The CB1 cannabinoid receptor mediates excitotoxicity-induced neural progenitor proliferation and neurogenesis. J. Biol. Chem..

[77] Berghuis P., Rajnicek A.M., Morozov Y.M., Ross R.A., Mulder J., Urban G.M., Monory K., Marsicano G., Matteoli M., Canty A., Irving A.J., Katona I., Yanagawa Y., Rakic P., Lutz B., Mackie K., Harkany T. (2007). Hardwiring the brain: endocannabinoids shape neuronal connectivity. Science.

[78] McPartland J.M., Skinner E. (2005). The biodynamic model of osteopathy in the cranial field. Explore (NY).

[79] Zhang F., Tsai S., Kato K., Yamanouchi D., Wang C., Rafii S., Liu B., Kent K.C. (2009). Transforming growth factor-beta promotes recruitment of bone marrow cells and bone marrow-derived mesenchymal stem cells through stimulation of MCP-1 production in vascular smooth muscle cells. J. Biol. Chem..

